# High-dimensional immune cell profiling of cerebrospinal fluid from patients with metastatic breast cancer and leptomeningeal disease

**DOI:** 10.1038/s41523-023-00526-1

**Published:** 2023-04-07

**Authors:** K. W. Im, L. A. Huppert, L. Malevanchik, H. S. Rugo, A. J. Combes, M. J. Campbell, M. F. Krummel, M. E. Melisko

**Affiliations:** 1 Department of Pathology and ImmunoX Initiative, University of California at San Francisco, San Francisco, CA 94143 USA; 2 UCSF CoLabs, University of California San Francisco, San Francisco, CA USA; 3 Division of Hematology/Oncology, Department of Medicine, University of California, San Francisco, San Francisco, CA USA; 4 Division of Hospital Medicine, Department of Medicine, University of California, San Francisco, San Francisco, CA USA; 5 Division of Gastroenterology, Department of Medicine, University of California, San Francisco, San Francisco, CA USA; 6 Department of Surgery, University of California San Francisco, San Francisco, CA USA

**Keywords:** Breast cancer, Cancer microenvironment, Cancer microenvironment

## Abstract

Leptomeningeal disease (LMD) is a devastating complication of metastatic breast cancer (MBC). In this non-therapeutic study, we enrolled 12 patients with MBC and known or suspected LMD who were undergoing a lumbar puncture as part of clinical care and collected extra cerebrospinal fluid (CSF) and a paired blood sample from each patient at a single time point. Of the 12 patients, 7 patients are confirmed to have LMD based on positive cytology and/or convincing MRI imaging (LMD^pos^), and 5 patients are deemed not to have LMD based on similar criteria (LMD^neg^). Using high-dimensional, multiplexed flow cytometry, we profile and compare the CSF and peripheral blood mononuclear cell (PBMCs) immune populations between patients with LMD and those without. Patients with LMD observe a lower overall frequency of CD45^+^ cells (29.51% vs. 51.12%, *p* < 0.05), lower frequencies of CD8^+^ T cells (12.03% vs. 30.40%, *p* < 0.01), and higher frequency of T_regs_ than patients without LMD. Interestingly, the frequency of partially exhausted CD8^+^ T cells (CD38^hi^TIM3^lo^) is ~6.5-fold higher among patients with LMD vs. those without (2.99% vs. 0.44%, *p* < 0.05). Taken together, these data suggest that patients with LMD may have lower overall immune infiltrates than patients without LMD, suggesting a more permissive CSF immune microenvironment but a higher frequency of partially exhausted CD8^+^ T cells, which may offer an important therapeutic target.

## Introduction

Patients with metastatic breast cancer (MBC) can develop metastases to the leptomeninges and subarachnoid space surrounding the brain and spinal cord, a condition known as a leptomeningeal disease (LMD). Approximately 5–10% of patients with MBC develop LMD during the course of their disease, causing significant morbidity and mortality^1,2^. The prognosis after the diagnosis of LMD is poor, with a median survival of 2–6 months, a statistic that has not changed significantly in over 20 years of published data^3,4^. More recently, there are reports of longer survival in patients with certain breast cancer subtypes with LMD, including those with HER2 positive MBC in which treatment with intrathecal (IT) HER2-directed therapies such as trastuzumab has resulted in median survival of closer to 12 months^5,6^. Patients with LMD are often excluded from clinical trials, and there are few effective treatments^1,2^. Therefore, it is important to better characterize the biology of this condition to develop better diagnostic and treatment modalities.

Historically, it was thought that the central nervous system (CNS) is an immune-privileged site, an organ that is relatively isolated from the influences of the immune system^7^. However, it is now well-established that immune cells are present in the brain and cerebrospinal fluid (CSF)^8,9^. There have been recent efforts to characterize the CSF immune profile in patients with LMD across tumor types. For example, in a study of patients with metastatic melanoma and CNS disease, Smalley et al. performed single-cell RNA sequencing on brain metastases and CSF samples and demonstrated that patients with LMD had a CSF microenvironment characterized by an immune-suppressed T-cell landscape distinct from that of brain and skin metastases^8^. In a study of patients with solid tumor malignancies and LMD receiving intravenous (IV) immune checkpoint inhibitors (ICIs), Prakadan et al. evaluated the CSF immune profiles before and after IV ICI treatment and found progressive CD8^+^ T cell abundance within the CSF^9^.

In addition to understanding the types of CSF immune cells, it is also important to characterize their functional state. Tumor-specific CD8^+^ T cells are exposed to persistent antigenic stimulation that leads to a dysfunctional state called “exhaustion,” which in turn leads to attenuated effector function and failure to control tumor progression^10–12^. A major goal of cancer immunotherapy is to reinvigorate exhausted and dysfunctional T cells to improve anti-cancer response. While the aforementioned studies described some characteristics of the CSF immune profiles of patients with LMD, the number of breast cancer patients in these studies was limited, and no published study to our knowledge has evaluated the exhaustion states of T cells in the CSF of patients with MBC and LMD.

Understanding the CSF immune profile may have therapeutic implications. For example, there has been recent interest in whether IV ICI therapy has CNS activity. Several studies evaluated the efficacy of IV pembrolizumab^13,14^ and the combination of IV nivolumab and ipilimumab^15^ in patients with solid tumor malignancies and LMD. However, it is unknown whether there is adequate CNS penetration of these agents, and it is possible that intrathecal (IT) administration may be superior. Therefore, IT ICI therapy has been proposed, with reports of using IT nivolumab^16,17^ and IT pembrolizumab^18^. However, efficacy in these studies is relatively limited, and understanding the immune milieu in the CSF is likely to provide important insights about how to improve these therapies for patients with CNS disease based on targeted hypotheses.

In this non-therapeutic study, we enrolled 12 patients with MBC and known or suspected LMD who were undergoing a lumbar puncture (LP) as part of clinical care and collected extra CSF and a paired blood sample from each patient at a single time point; there were no restrictions on breast cancer subtype or lines of prior therapies in this pragmatic study. Of the 12 patients, 7 patients were ultimately confirmed to have LMD based on positive CSF cytology and/or convincing MRI imaging^19^ (LMD^pos^), and 5 patients were deemed not to have LMD based on similar criteria (LMD^neg^). Using a high-dimensional, multiplexed flow cytometric screen to profile immune cell populations, we evaluated the immune cell profiles in the CSF and peripheral blood mononuclear cell (PBMC) samples and compared the immune cell landscape between patients with LMD (LMD^pos^) versus those without LMD (LMD^neg^).

## Results

### Participant characteristics and treatment history

**Fig. 1:**
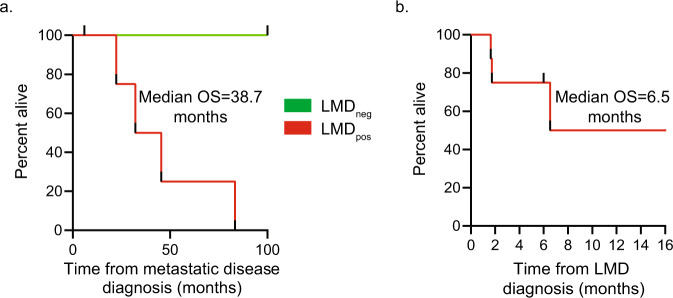
Median overall survival from time of metastatic disease and LMD diagnosis. **a** Median overall survival from the time of metastatic disease diagnosis among patients with LMD (red) and those without LMD (green). **b** Median overall survival from the time of LMD diagnosis among the patients with LMD in months.

Twelve patients with MBC and known or suspected LMD were enrolled in this study: 7 patients were ultimately confirmed to have LMD based on positive CSF cytology and/or convincing MRI imaging (LMD^pos^), and 5 patients did not have LMD based on similar criteria (LMD^neg^). Demographic characteristics, tumor characteristics, treatment history, and CNS involvement details are shown in Table 1. Among patients with LMD, 4 had ductal histology (57.1%), and 3 had lobular (42.9%); 5 had estrogen receptor-positive (ER+), human epidermal growth factor receptor 2 negative (HER2−) disease (71.4%) and 2 had ER+/HER2+ disease (28.6%). Among patients without LMD, 4 had ductal histology (80.0%), and 1 had lobular (20.0%); 1 had ER+/HER2− disease (20.0%), 3 had ER+/HER2+ disease (60.0%), and 1 had ER−/HER2+ disease. Most patients in both groups were heavily pre-treated, with 3 median lines of therapy in the metastatic setting among patients with LMD (range 0–12) and 2 median lines of therapy in the metastatic setting among patients without LMD (range 2–6). Median overall survival from the time of metastatic disease diagnosis was 38.7 months among patients with LMD; all patients without LMD were alive at the time of data cutoff (Fig. 1a). Among patients with LMD, median overall survival from the time of LMD diagnosis to death was 6.5 months (Fig. 1b).

**Table 1:** Patient demographics and treatment history.

Leptomeningeal disease status	LMD^pos^ (*n* = 7)	LMD^neg^ (*n* = 5)
*Demographic characteristics*		
Sex		
Female	7 (100.0%)	5 (100.0%)
Male	0 (0.0%)	0 (0.0%)
Median age, years (range)	62.3 (49.7–71.3)	61.8 (47.0–70.1)
*Tumor characteristics*		
Tumor histology		
Ductal	4 (57.1%)	4 (80.0%)
Lobular	3 (42.9%)	1 (20.0%)
*Receptor status*		
ER+/HER2−	5 (71.4%)	1 (20.0%)
ER+/HER2+	2 (28.6%)	3 (60.0%)
ER−/HER2+	0 (0.0%)	1 (20.0%)
TNBC	0 (0.0%)	0 (0.0%)
*De novo metastatic disease*		
Yes	2 (28.6%)	0 (0.0%)
No	5 (71.4%)	5 (100.0%)
*Treatment history in the metastatic setting*		
Median lines of therapy in the metastatic setting prior to CSF sample collection		
Endocrine therapy, median lines (range)	1 (0–3)	1 (0–1)
Chemotherapy, median lines (range)	1 (0–8)	1 (0–5)
Total lines of therapy (range)	3 (0–12)	2 (2–6)
*Intrathecal therapy prior to CSF sample collection*		
Yes^c^	4 (57.1%)	0 (0.0%)
No	3 (42.9%)	5 (100.0%)
*CNS radiation therapy prior to CSF sample collection*		
Yes	6 (85.7%)	1 (20.0%)
No	1 (14.3%)	4 (80.0%)
*Central nervous system disease*		
Brain metastases		
Yes	5 (71.4%)	3 (60.0%)
No	2 (28.6%)	2 (40.0%)
*Leptomeningeal disease diagnosis*		
CSF cytology		
Positive for malignant cells	5 (71.4%)	0 (0.0%)
Negative for malignant cells	2 (28.6%)	5 (100.0%)
*MRI brain/spine*		
Positive for evidence of LMD	7 (100.0%)	0 (0.0%)
Equivocal for evidence of LMD	0 (0.0%)	3 (60.0%)
Negative for evidence of LMD	0 (0.0%)	2 (40.0%)

*LMD* leptomeningeal disease, *ER* estrogen receptor, *HER2* human epidermal receptor 2 negative, *TNBC* triple-negative breast cancer, *MRI* magnetic resonance imaging.

### CSF immune profiling demonstrates decreased frequencies of CD8^+^ T cell populations in the CSF of patients with LMD vs. those without LMD

**Fig. 2:**
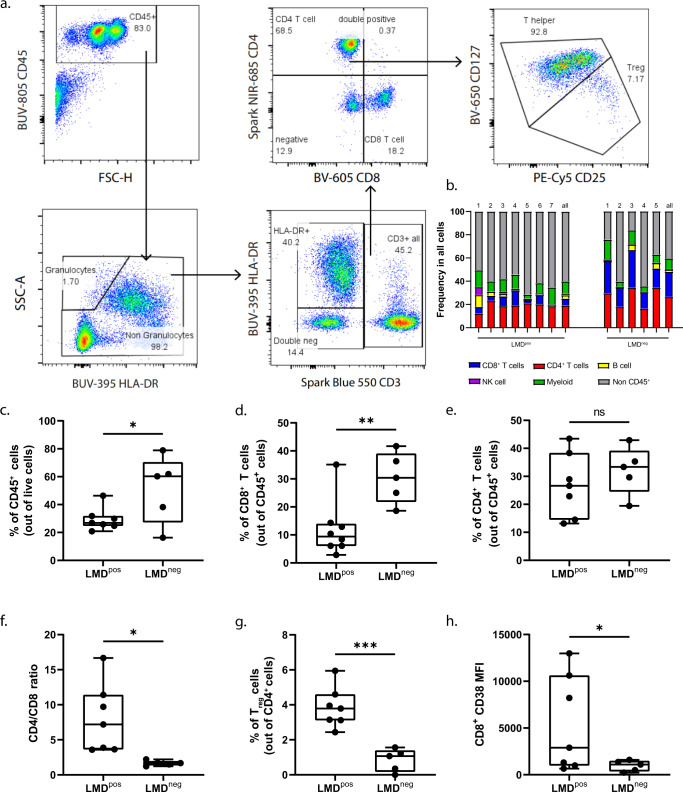
Decreased frequencies of CD8^+^ T cell populations in the CSF of patients with leptomeningeal disease. **a** Gating strategy to identify leukocyte frequencies pre-gated from singlets and live populations in CSF. **b** Frequency plot in all cells of each individual patient and cumulative mean (all) comparing LMD^pos^ (left columns) and LMD^neg^ (right columns) in CSF. **c**–**e** Box plots comparing the frequency of immune cells between LMD^pos^ vs. LMD^neg^ by the following immune cell types: **c** CD45^+^ cells, **d** CD8^+^ T cells, and **e** CD4^+^ T cells. **f** CD4/CD8 T cell ratios and **g** frequency of T_reg_. (T_reg_ populations were defined as CD4^+^CD25^+^CD127^lo^). **h** Median fluorescent intensity of CD38 expression on CD8^+^ T cells. For box plots in this figure, the boxes indicate the interquartile range, while the lower and upper bars correspond to the minimum and maximum non-outlier values of the data distribution. Centerline indicates the median value. Statistical significance was assessed in **c**–**h** by unpaired student *t* test. (* or **) indicates statistical significance with *p* < 0.05, *p* < 0.01 respectively. n.s not significant.

To profile the CSF and PBMC immune microenvironments, we utilized high dimensional flow cytometry using a marker panel that included a broad array of immune cell markers^20^, including markers of exhaustion and function (Supplementary Fig. 1). First, we evaluated the CSF immune profile via conventional flow gating to identify immune cell populations and their distribution between patients in the CSF (Fig. 2a, b). The frequency of total CD45^+^ and CD8^+^ T cells was significantly lower in patients with LMD^pos^ compared to LMD^neg^ (29.51% vs. 51.12%, *p* < 0.05, Fig. 2c; 12.03% vs. 30.40%, *p* < 0.01, Fig. 2d, respectively). There were no statistically significant differences in the frequency of CD4^+^ T cells or CD14^+^ myeloid cells (defined as HLA-DR^+^CD14^+^CD3^−^CD19^−^) between groups (Fig. 2e and Supplementary Fig. 2a, b). Next, we evaluated whether the lower frequency of CD8^+^ T cells in patients with LMD may be related to changes in overall T cell frequencies. Indeed, CD4^+^/CD8^+^ T cell ratios were increased ~6-fold in LMD^pos^ vs. LMD^neg^ (8.01 vs. 1.66, *p* < 0.05, Fig. 2f. Moreover, the frequency of T regulatory (T_reg_) cells was ~3-fold higher in LMD^pos^ compared to LMD^neg^ (3.82 vs. 0.84, *p* < 0.01, Fig. 2g. We also evaluated each T cell profile based on their exhaustion phenotype. We did not detect any differences in the expression of PD-1, LAG3, and TIM3 in CD8^+^ T cells between groups (Supplementary Fig. 2c–e). Interestingly, CD38 expression was significantly increased in LMD^pos^ vs. LMD^neg^ (5797 vs. 959.60 MFI, *p* < 0.05, Fig. 2h). Although increases in CD4^+^/CD8^+^ T and frequency of T_reg_ were observed in LMD^pos^_,_ no differences were detected in the exhaustion phenotypes of CD4^+^ T cells between groups (data not shown). Overall, we noted big differences in the immune landscape of LMD^pos^. Specifically, CD38^+^ expression on CD8^+^ T cells was observed to be higher than LMD^neg^ in the CSF. These initial findings led us to investigate the relationship between each exhaustion marker in T cells between groups.

### High-dimensional immune cell profiling in the CSF reveals distinct CD8^+^ T and myeloid cell phenotypes in patients with LMD vs. those without LMD

**Fig. 3:**
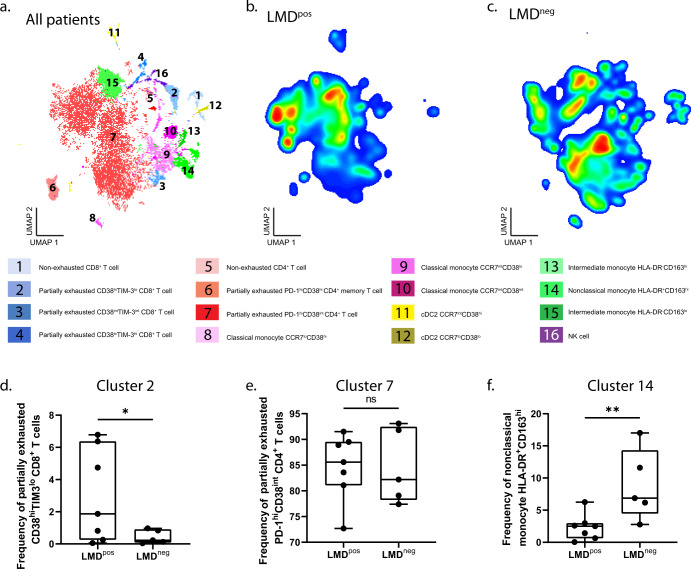
High-dimensional immune cell profiling in CSF reveals distinct CD8^+^ T and myeloid cell phenotypes. **a** UMAP projection of annotated clusters from all patients. **b**, **c** Density heat maps of immune cells in patients with LMD (LMD^pos^) (**b**) and patients without LMD (LMD^neg^) (**c**), with red representing the most prevalent cell types and blue representing the least prevalent cell types. **d** Frequency box plots of cluster 2, partially exhausted CD8^+^ T cell (CD38^hi^TIM3^lo^), **e** cluster 7, partially exhausted CD4^+^ T cells (PD-1^hi^CD38^int^), and **f** cluster 14 nonclassical monocytes expressing HLA-DR and CD163^hi^. For box plots in this figure, the boxes indicate the interquartile range, while the lower and upper bars correspond to the minimum and maximum non-outlier values of the data distribution. Centerline indicates the median value. Statistical significance was assessed in **d**–**f** by one-way ANOVA followed by the Tukey post hoc test. (* or **) indicates statistical significance with *p* < 0.05, *p* < 0.01 respectively. n.s not significant.

T exhausted cells initially undergo T cell activation programs that include the downregulation of naïve markers such as CCR7 while activation markers such as CD38 are induced^21–23^. Given the advantage of our high-dimensional marker panel to assess immune cell populations and uncover multiparametric features, we performed an unsupervised dimensionality reduction analysis to obtain a more detailed assessment of immune subsets present in the CSF of patients with and without LMD. Dimensionality reduction (UMAP) and hierarchical clustering identified 16 populations of cell phenotypes (Fig. 3a) Densities of these 16 identified clusters differed between LMD^pos^ (Fig. 3b) and LMD^neg^ (Fig. 3c). All four clusters observed in CD8^+^ T cells were low in CCR7^+^ expression with varying levels of PD-1, TIM3, LAG3, and CD38 (Supplementary Fig. 3a). Since none of these clusters had high expression of all markers to indicate terminal exhaustion, clusters 2–4 were annotated as partially exhausted CD8^+^ T cell clusters. Additionally, partially exhausted CD8^+^ T cells (cluster 2: defined as CD38^hi^TIM3^lo^) were ~6.5-fold higher among LMD^pos^ vs. LMD^neg^ (2.99% vs. 0.44%, *p* < 0.05, Fig. 3d) while other clusters 1, 3, and 4 remain unchanged (Supplementary Fig. 3b–d). There were no differences in the frequency of partially exhausted CD4^+^ T cells (defined as PD-1^hi^CD38^int^) (Fig. 3e) or non-exhausted CD4^+^ T cells (defined as PD-1^lo^, LAG3^lo^, TIM3^lo^, CD38^lo^) (data not shown). Finally, the frequency of nonclassical monocytes expressing HLA-DR^+^CD163^hi^ was ~3.4-fold lower in LMD^pos^ vs. LMD^neg^ (2.59% vs. 8.89%, *p* < 0.01, Fig. 3f). Taken together, high-dimensional profiling in the CSF in patients with LMD revealed differences in CD8^+^ T cell exhaustion and myeloid phenotypes in patients with LMD compared to those without LMD.

### PBMC immune profiling indicates few changes in coarse leukocyte populations in patients with LMD vs. those without LMD

**Fig. 4:**
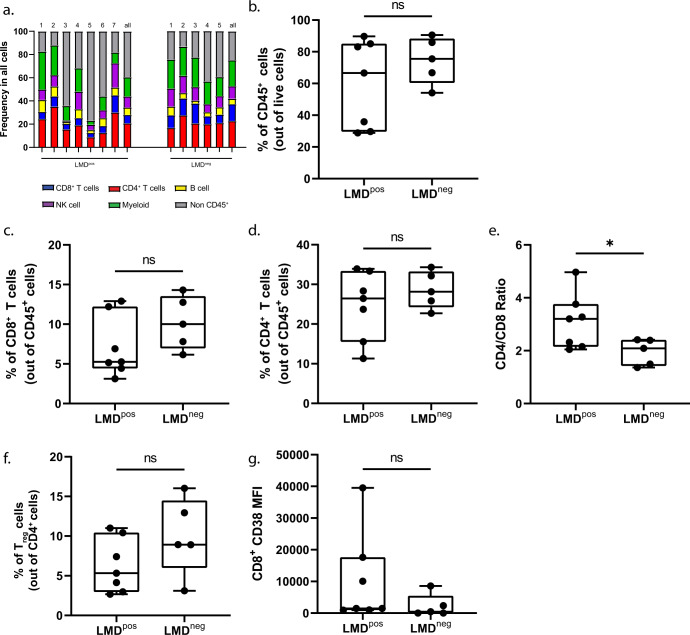
Coarse changes in leukocyte populations in PBMCs are minimal. **a** Frequency plot in all cells of each individual patient and cumulative mean (all) comparing LMD^pos^ (left columns) and LMD^neg^ (right columns) in PBMCs. **b**–**d** Box plots comparing the frequency of immune cells between LMD^pos^ vs. LMD^neg^ by the following immune cell types: **b** CD45^+^ cells, **c** CD8^+^ T cells, **d** CD4^+^ T cells. **e** CD4/CD8 T cell ratios and **f** frequency of T_reg_. **g** Median fluorescent intensity of CD38 expression on CD8^+^ T cells. For box plots in this figure, the boxes indicate the interquartile range, while the lower and upper bars correspond to the minimum and maximum non-outlier values of the data distribution. Centerline indicates the median value. The gating strategy for this figure is derived from Fig. 2a. Statistical significance was assessed in **b**–**g** by unpaired student *t*-test. (*) indicates statistical significance with *p* < 0.05, *p* < 0.01 respectively. n.s not significant.

Next, we investigated whether unique immune profiles could also be detected in the PBMC fraction of patients with LMD vs. those without, similar to what was seen in the CSF samples. In this analysis, we found no differences in the frequencies of CD45^+^ cells, CD8^+^ T cells, CD4^+^ T cells, or myeloid cells between patients with LMD vs. those without (Fig. 4a–d, Supplementary Fig. 4a). Similar to CSF, CD4/CD8 T cell ratios were increased in LMD^pos^ vs. LMD^neg^ (3.20 vs. 1.79, *p* < 0.05, Fig. 4e). However, there were no differences between groups in terms of the frequency of T_reg_ and CD38 expression in CD8^+^ T cells in the PMBCs (Fig. 4f, g).

### High-dimensional immune profiling in the PBMC fraction reveals unique subsets of CD4^+^ T cell and monocyte populations that differ between patients with LMD and those without LMD

**Fig. 5:**
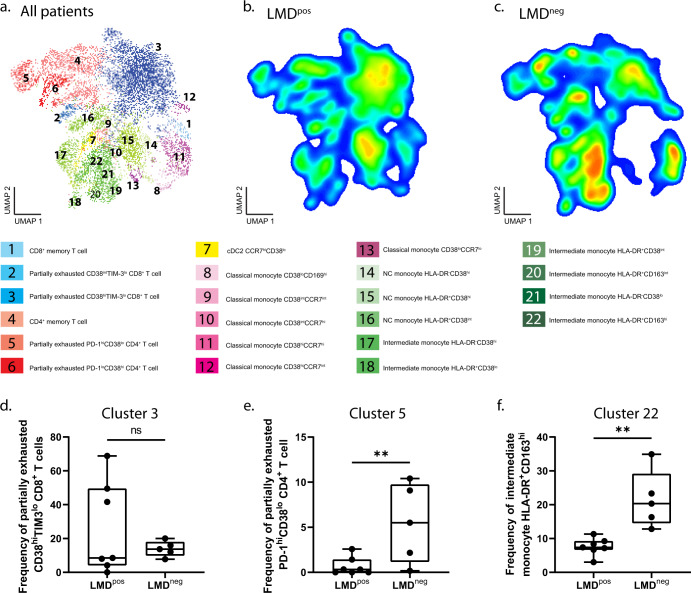
Unsupervised immune profiling in PBMCs reveals unique subsets of CD4^+^ T cell and monocyte populations. **a** UMAP projection of annotated clusters from all patients. **b**, **c** Density heat maps of immune cells in patients with LMD (LMD^pos^) (**b**) and patients without LMD (LMD^neg^) (**c**), with red representing the most prevalent cell types and blue representing the least prevalent cell types. **d** Frequency box plots of cluster 3, partially exhausted CD8^+^ T cell (CD38^hi^TIM3^lo^), **e** cluster 5, partially exhausted CD4^+^ T cell (PD-1^hi^CD38^lo^), and **f** cluster 22, intermediate monocytes expressing HLA-DR and CD163 out of the 22 identified cluster populations. For box plots in this figure, the boxes indicate the interquartile range, while the lower and upper bars correspond to the minimum and maximum non-outlier values of the data distribution. Centerline indicates the median value. Statistical significance was assessed in **d**–**f** by one-way ANOVA followed by the Tukey post hoc test. (* or **) indicates statistical significance with *p* < 0.05. n.s not significant.

We then performed high-dimensional immune profiling with the PMBC samples (Supplementary Fig. 4b) and identified 22 myeloid and lymphoid clusters (Fig. 5a), with different profiles in LMD^pos^ (Fig. 5b) vs. LMD^neg^ (Fig. 5c). Interestingly, there were no NK or B cells defined as a cluster from LMD^pos^ and LMD^neg^ as opposed to healthy controls observing all conventional immune cell types (Supplementary Fig. 5a, b). There were no differences in the frequency of cluster 3 partially exhausted CD8^+^ T cells (CD38^hi^TIM3^lo^) between groups (Fig. 5d). However, the frequency of cluster 5 partially exhausted CD4^+^ T cells (PD-1^hi^CD38^lo^) was significantly lower in LMD^pos^ vs. LMD^neg^ (0.72 vs. 5.45, *p* < 0.05, Fig. 5e). When observing myeloid clusters, a lower frequency of cluster 22 intermediate monocytes expressing HLA-DR^+^CD163^hi^ in LMD^pos^ vs. LMD^neg^ (7.37 vs. 18.30, *p* < 0.01, Fig. 5f) was observed.

## Discussion

In this non-therapeutic study, we used high-dimensional, multiplexed flow cytometry to profile and compare the CSF and PBMC immune cell populations between patients with MBC and LMD vs. those without LMD. These data suggest some differences in the PBMC immune profiles between patients with LMD vs. those without, although these differences were less striking than those in the CSF immune profiles. Previous studies that characterized the CSF immune profiles in patients with LMD observed predominant proportions of CD4^+^ T, CD8^+^ T, and myeloid cell clusters at the transcriptomic level^8,9,24^. Our findings support and expand upon these observations by investigating functional markers on these immune cell populations at the proteomic level and especially markers associated with T cell exhaustion^25–27^.

In this study, we observed that patients with LMD had lower overall frequencies of CSF CD45^+^ cells, lower frequencies of CD8^+^ T cells, and higher frequencies of T_reg_ than patients without LMD, suggesting a more permissive CSF immune microenvironment in patients with LMD compared to those without LMD. It is possible that these differences in immune profiles are due to differences in prior or current treatments, but notably, median lines of treatment were similar between groups. It is unclear if this relative decrease in immune infiltrates preceded the development of LMD, thus allowing tumor cells to survive in the CSF microenvironment, or if, instead, this is a consequence of malignant infiltration into that space. Future research with serial CSF samples over time, including time points prior to the development of LMD, would be useful to clarify this question.

Conventional markers of T cell exhaustion are PD-1, TIM3, and LAG3^22^. However, recent evidence suggests that CD38 is a mechanism of acquired resistance to PD-1/PD-L1 blockade, leading to CD8^+^ T cell exhaustion^23,26^ and lower survival in patients with kidney cancer^28^. Despite lower overall frequencies of immune infiltrates in the CSF of patients with LMD, there was a subset of CD8^+^ T cells (CD38^hi^TIM3^lo^) that were enriched. Given that this population did not express high levels of PD-1 or LAG3, we characterized these as partially exhausted^22^. It is possible that targeting these T cells may be a strategy for improving immunogenicity against malignant cells in the CSF, although further functional studies are needed to clarify the functional role of these cells and to characterize how these and other T cell clusters in the CSF and PBMC fraction evolve over time. Taken together, these findings have potential therapeutic implications, as a major principle of action of cancer immunotherapy with immune checkpoint inhibitors is to reinvigorate exhausted and/or dysfunctional T cells^29–32^, so it may be possible to use ICI or other therapies to reinvigorate these cells and provide therapeutic benefit.

Circulating monocytes are a major source of tumor-associated macrophages (TAMs) in breast cancer. In addition to differences seen in T cell populations, we also observed differences in a population of monocytes that expressed HLA-DR and CD163 that was less abundant in both the CSF and PBMC samples of LMD^pos^ vs. LMD^neg^. While it is still unknown, we hypothesize that these circulating monocytes play a critical role in influencing the tumor microenvironment, specifically T cell exhaustion states. Previous studies reported that CD163 expression might be a prognostic biomarker and predictor of overall survival in patients with colorectal cancer^33,34^ and is also correlated with response to neoadjuvant chemotherapy in patients with early stage breast cancer^35^. In a mouse model of breast cancer, the onset of T cell exhaustion was linked to TAM abundance, and TAMs forming long-lasting synapses with CD8^+^ T cells further primes T cell exhaustion^36^. Taken together, the relationship between our identified population of CD163^+^ monocytes and TAMs in the CSF would need to be better characterized in future studies.

There are several important limitations of this work. First, to maximize CSF acquisition, this study included flexible inclusion criteria, allowing patients with any hormone and HER2 receptor subtype and without limitation on prior or current treatment history, including several patients with LMD who had received prior IT therapy. Despite these flexible inclusion criteria, there was a relatively similar distribution of breast cancer receptor subtypes and similar median overall lines of therapy among patients with LMD vs. those without LMD that were included in this analysis. Second, we only collected CSF specimens at a single time point in this study, given that many of these patients did not live long enough for serial sampling; in the future, we will attempt to collect serial samples in a subset of patients if possible. Third, immune cell profiles were analyzed by high dimensional flow cytometry using a select panel of markers, which may not fully capture the immune cell diversity. Future studies could expand the total number of markers to include additional functional markers like effector vs. naïve markers, interferon-gamma, and others^9,22^. In addition, single-cell omic approaches could support the validation and expansion of these findings^37,38^.

In conclusion, these data indicate that patients with MBC and LMD had a lower overall frequency of CD45^+^ and CD8^+^ T cells and a higher frequency of T_reg_ than patients without LMD, suggesting a more permissive immune environment and a higher frequency of partially exhausted CD8^+^ T cells (CD38^hi^TIM3^lo^). Larger studies are needed to validate these findings and determine whether there are prognostic or predictive features of the CSF immune cell profile which may have clinical utility. If one or more of these immune signatures is validated in a larger cohort, it is possible that it could serve as a biomarker to promote early detection, provide prognostic information, and inform treatment decisions for patients with MBC and LMD.

## Methods

### Participant identification and enrollment

Male or female patients ≥18 years who had histologically confirmed MBC with known or suspected LMD based on either radiographic or clinical findings and who were undergoing an LP for clinical care were eligible to enroll in this non-therapeutic study to collect extra CSF for research purposes. The study was approved by the University of California San Francisco (UCSF) Comprehensive Cancer Center Protocol Review Committee on Human Research, and written informed consent was obtained from all patients prior to trial enrollment. We also consented to five healthy controls who donated blood for research purposes to verify known immune cell populations in the PMBC fraction; these healthy controls were enrolled by the UCSF Immunoprofiler Initiative under a UCSF IRB-approved protocol.

### LMD diagnosis

The diagnosis of LMD was based on positive CSF cytology and/or convincing MRI imaging using standard criteria for the diagnosis of LMD^19^.

### Chart extraction

Detailed information about patient demographic characteristics, tumor histology, sites of metastatic involvement, treatment history, and survival status were obtained via manual chart extraction from the electronic medical record.

### CSF fluid extraction

CSF was extracted via an LP or tap of an Ommaya reservoir as part of clinical care. An extra 6–10cc of CSF was collected for this study into EDTA tubes (BD, 366643), stored on ice, and processed within 3 h of sample collection. CSF was spun at 1500 rpm for 15 min at 4 °C, and the supernatant was aspirated to collect the cell pellet for flow staining. CSF cell count and diff indicated scant red blood cell contamination (0–5 RBCs) in the CSF specimens, so red blood cell lysis protocol was not utilized for CSF specimens.

### Blood serum collection

A 5–10cc of serum was collected into EDTA tubes at the same time as the CSF fluid extraction, stored on ice, and processed within 3 hours of sample collection. Whole blood was prepared by treatment of 1 ml of peripheral blood with red blood cell lysis buffer (Roche, 11-814-389-001) according to the manufacturer’s instructions. PBMCs were then processed further for flow staining.

### Flow cytometry for CSF and PBMC samples

After cell isolation, CSF and PBMCs were stained with live/dead Aqua (Thermo #L23105) in phosphate buffer saline (PBS) for 15 min at 4 °C in the dark. After FACS (2% fetal calf serum, 0.05% Sodium azide, in PBS) buffer washes, cells were incubated with Human FcX (Biolegend #422302) for 10 min to prevent non-specific antibody binding. Following the Fc block, cells were stained with a master mix of CD45 BD Bio #612891; CD3 Biolegend #344852; CD4 Biolegend #344658; CD8 BD Bio #740411; CD14 BD Bio #740773; CD16 BD Bio #612944; CD19 antibodies.com a18909; CD38 BD Bio #564979; CD1c Biolegend #331516; BDCA3 BD Bio #565084; CD123 Invitrogen 62-1239-42; CD11c BD Bio #747459; CD197 Biolegend #353208; HLA-DR BD Bio #564040; PD-1 BD Bio #563789; CD127 BD Bio #563225; CD25 Invitrogen #15-0259-42; CD163 Biolegend #333610; CD223 Invitrogen #46-2239-42; CD366 Invitrogen #12-3109-42; CD56 Biolegend #392428; HER2 Biolegend #324412; CD169 Biolegend #346016. Cells were then washed and analyzed on the Cytek Aurora (Cytek Biosciences, Inc.) high dimensional flow cytometry machine. Our marker panel is also shown in Supplementary Fig. 1. Compensation was performed on the CyTEK Aurora by titrating each antibody individually and then assessing spectrograms for all markers added together. Then, spectral unmixing was performed to calculate compensation, and gating for cell populations was determined by fluorescent minus one (FMOs) and healthy PBMC donor cells.

### Uniform manifold approximation and projection (UMAP) visualization and computational analysis of flow cytometric data

All CSF or PBMC samples were analyzed by FloJo (v10.7.1), batch-corrected using CytoNorm (v1.2.3) from FloJo plugins, and concatenated to create a single, 100,000-cell composite file. In this composite file, cells were pre-gated as follows: singlets, live, and CD45^+^. A UMAP algorithm for dimensionality reduction was then applied using FloJo UMAP plugin (v3.1) for KNN = 30. Computational analysis was carried out on FlowSOM (v3.0.18) to delineate the total number of clusters generated, followed by ClusterExplorer (v1.6.5) and Morpheus (Broad Institute) for cell cluster annotations. All frequencies of clusters in bar plots delineated are calculated by dividing cluster “x” over all clusters. Hierarchical clustering was generated by one minus Pearson correlation on rows and columns, and expressions are transformed by subtracting row mean, divided by row standard deviation.

### Statistics and data visualization

All data were analyzed using Prism Software (GraphPad v9.3.1; San Diego, CA). Descriptive statistics were used to summarize numeric responses as the rate of events (%), median (95% confidence interval), or mean ± standard error mean as appropriate. The time to onset of metastases, time to LMD diagnosis and survival distributions was estimated by the Kaplan–Meier non-parametric method. Frequency bar plot statistics were performed by unpaired *t*-tests between groups for coarse assessment of immune populations. Frequency bar plots for comparing multiple clusters from UMAP between patients with or without LMD were generated by performing one-way ANOVA followed by the Tukey post hoc test. All data on specific statistical tests and resulting significance levels can be found in each figure legend. Data collection and analysis of immune profiles from groups were performed blind until endpoint validation by clinical chart information. All figures were generated with GraphPad Prism and curated in Adobe Illustrator.

### Institutional review board statement

This research was approved by the UCSF Institutional Review Board.

## Supplementary information

Supplementary Figures

## Data Availability

For clinical research purposes, patient data is anonymized. please contact the corresponding authors to provide additional materials or data upon request.
