# Supplementary material for: High-dimensional immune cell profiling of cerebrospinal fluid from patients with metastatic breast cancer and leptomeningeal disease
Source: NPJ Breast Cancer. 2023 Apr 7;9:22. doi: 10.1038/s41523-023-00526-1 (PMC10082042; doi:10.1038/s41523-023-00526-1)
Supplement: Supplementary file 1 — Supplementary Figures [file 41523_2023_526_MOESM1_ESM.pdf]

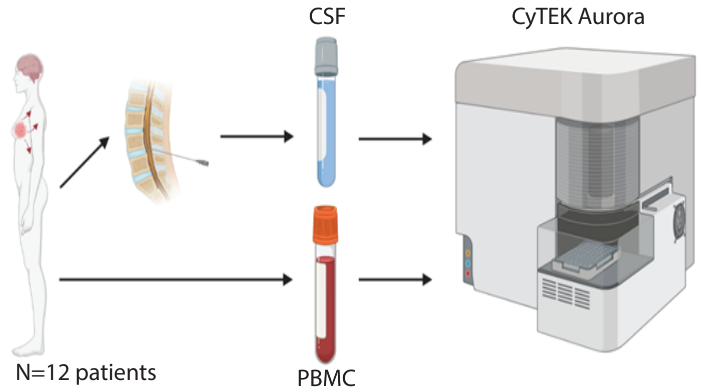

Approximate Emission Wavelength (nm	UV 355nm		Violet 405nm		Blue 488 nm		YG 561 nm		Red 640 nm	
	Antigen	Fluor	Antigen	Fluor	Antigen	Fluor	Antigen	Fluor	Antigen	Fluor
380	HLADR	BUV395								
400										
420			CD197	BV421						
440			CD123	SB436						
450	Live	Via UV Blu								
480										
500	CD16	BUV496			BDCA3	BB515				
510										
520										
550			CD19	Pac O	CD3	Sprk B 550				
570							TIM3	PE		
580										
600			CD8	BV605			CD169	PEDazzle		
610										
620										
650			CD127	BV650			CD25	PECy5	CD163	APC
665										
680									HER2	AF647
690									CD4	Spark NIR 685
700							CD56	PEFire700	CD38	APCR700
710			CD14	BV711	LAG3	PerCPefl710				
740										
750			CD11c	BV750						
780			PD1	BV786			BDCA1	PECy7	CD45RO	APCefluor780
800	CD45	BUV805								

	Antibody	Fluorochrome
Pan-leukocyte marker	CD45	BUV805
Exhaustion markers	PD-1	BV786
	TIM3	PE
	LAG3	PerCP-efluor710
	CD38	APC-R700
Myeloid markers	CD14	BV711
	CD16	BUV496
	CD11c	BV750
	CD123	Super Bright 436
	BDCA1	PE-Cy7
	BDCA3	BB515
T cell markers	CD3	Spark Blue 550
	CD4	Spark NIR
	CD25	PE-Cy5
	CD127	BV650
	CD8	BV605
Functional markers	HLA-DR	BUV395
	CD163	APC
	CD169	PE-Dazzle
	CD197	BV421
B cell marker	CD19	Pacific Orange
NK marker	CD56	PE-Fire700

Supplementary Figure 1. *Study schema and marker panel for immune profiling CSF and PBMCs* Patients with metastatic breast cancer and known or suspected leptomeningeal disease were eligible to enroll in this study. Participants underwent a lumbar puncture or Ommaya tap to collect excess cerebrospinal fluid (blue) as well as paired research blood sample for peripheral blood mononuclear cell (PBMCs) isolation (red). The CSF and PBMC immune cell characteristics were analyzed by a high dimensional flow cytometry screen using the markers associated with cell phenotype, exhaustion, and functional markers. Schematic diagram was created from Biorender.com and marker designation to channel spreadsheet was created from Parnassus Flow Cytometry Core.

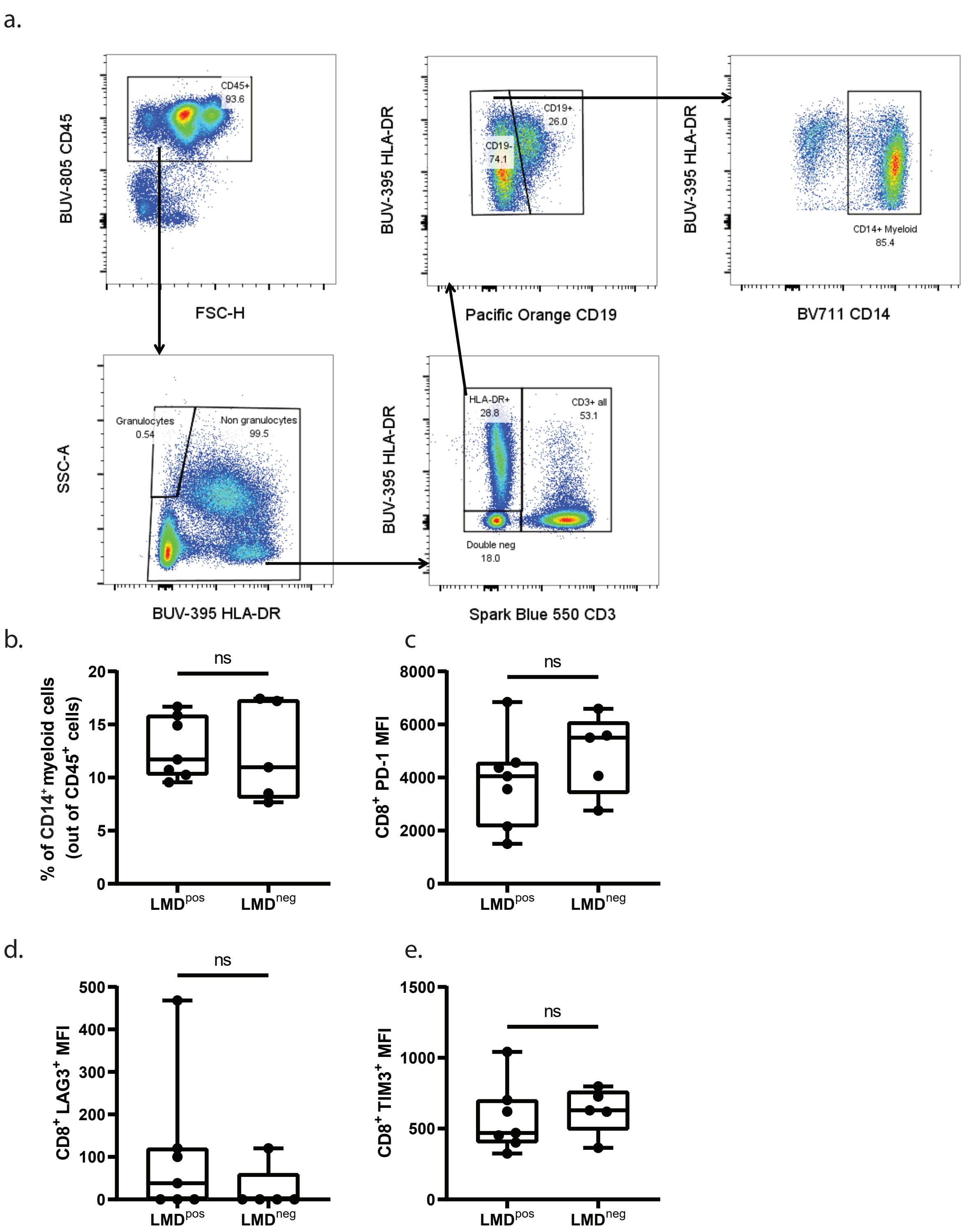

Supplementary Figure 2. *CSF myeloid frequency and exhaustion markers on CD8$^+$ T cells*
a) Gating strategy to identify CD14$^+$ myeloid frequencies pre-gated from singlets and live populations in CSF and PBMCs presented on Supplementary Fig 4a. **b**) Frequency box plot of myeloid cells (defined as HLA-DR$^+$CD14$^+$CD3$^-$CD19$^-$) between LMD^{pos} vs. LMD^{neg} groups in CSF. **c-e**) Box plots on median fluorescent intensities in CD8$^+$ T cell populations for c) PD-1, **d**) LAG3, and **e**) TIM3. For box plots in this figure, the boxes indicate the interquartile range, while the lower and upper bars correspond to the minimum and maximum non-outlier values of the data distribution. Center line indicates the median value. Statistical significance was assessed in **b-e** by unpaired student t test. n.s not significant.

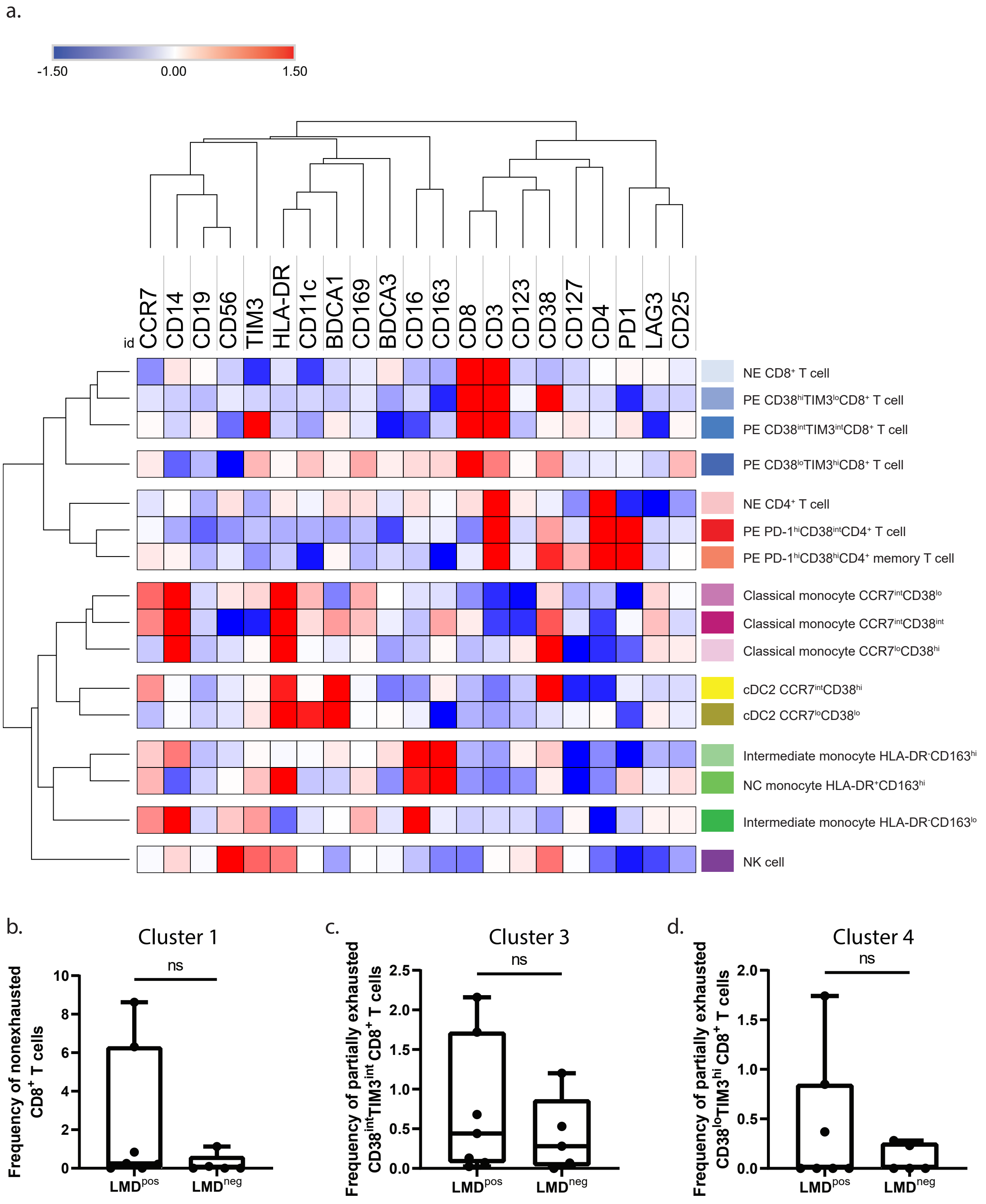

Supplementary Figure 3. *CSF unsupervised clustering heatmap and frequency of $CD8^+$ T cell clusters* **a**) Generated heatmap of marker panel expression and hierarchical clustering for cluster annotation in CSF. Blue denotes lower expression while red denotes higher expression of each marker within a cluster (top scale bar, left to right). Hierarchical clustering was performed by one minus Pearson correlation. Frequency box plots of $CD8^+$ T cell clusters for: **b**) Cluster 1, non-exhausted $CD8^+$ T cells, **c**) Cluster 3, $CD38^{int}TIM3^{int}$ partially exhausted $CD8^+$ T cells, **d**) Cluster 4, $CD38^{lo}TIM3^{hi}$ partially exhausted $CD8^+$ T cells. Statistical significance was assessed in **b-d** by one-way ANOVA followed by tukey post hoc test. n.s, not significant.

a.

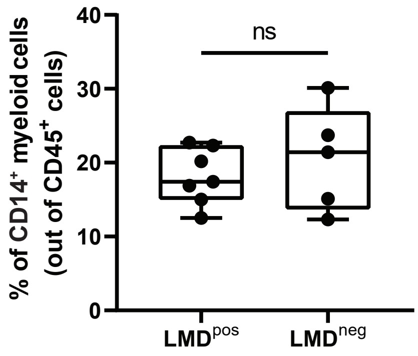

b.

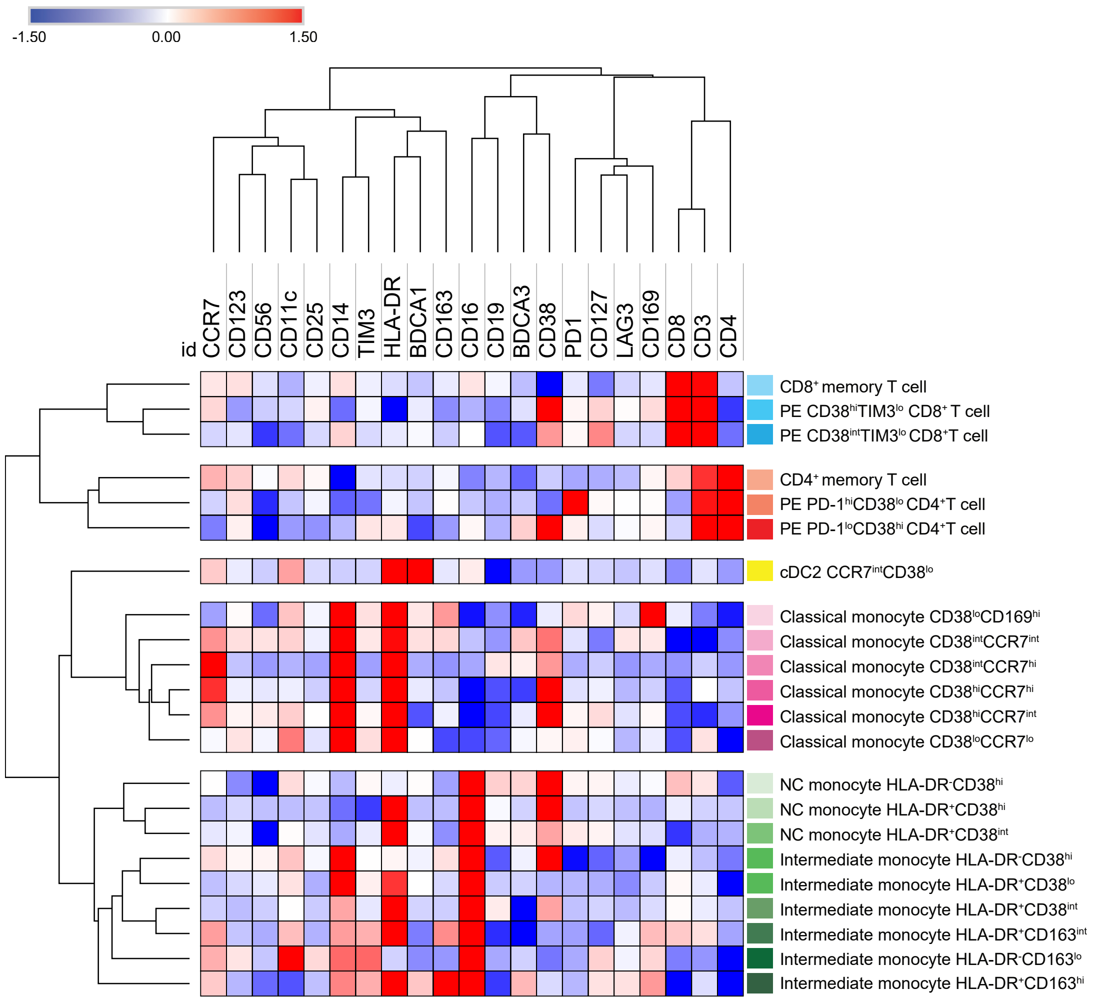

Supplementary Figure 4. *PBMC myeloid frequency and unsupervised clustering heatmap*

a) Frequency box plot of $CD14^{+}$ myeloid cells between LMD^{pos} vs. LMD^{neg} groups in PBMC. Statistical significance was assessed by unpaired student t test. Gating strategy is derived from Supplementary Figure 2a. **b**) Generated heatmap of marker panel expression and hierarchical clustering for cluster annotation. Blue denotes lower expression while red denotes higher expression of each marker within a cluster (top scale bar, left to right). Hierarchical clustering was performed by one minus Pearson correlation. n.s not significant.

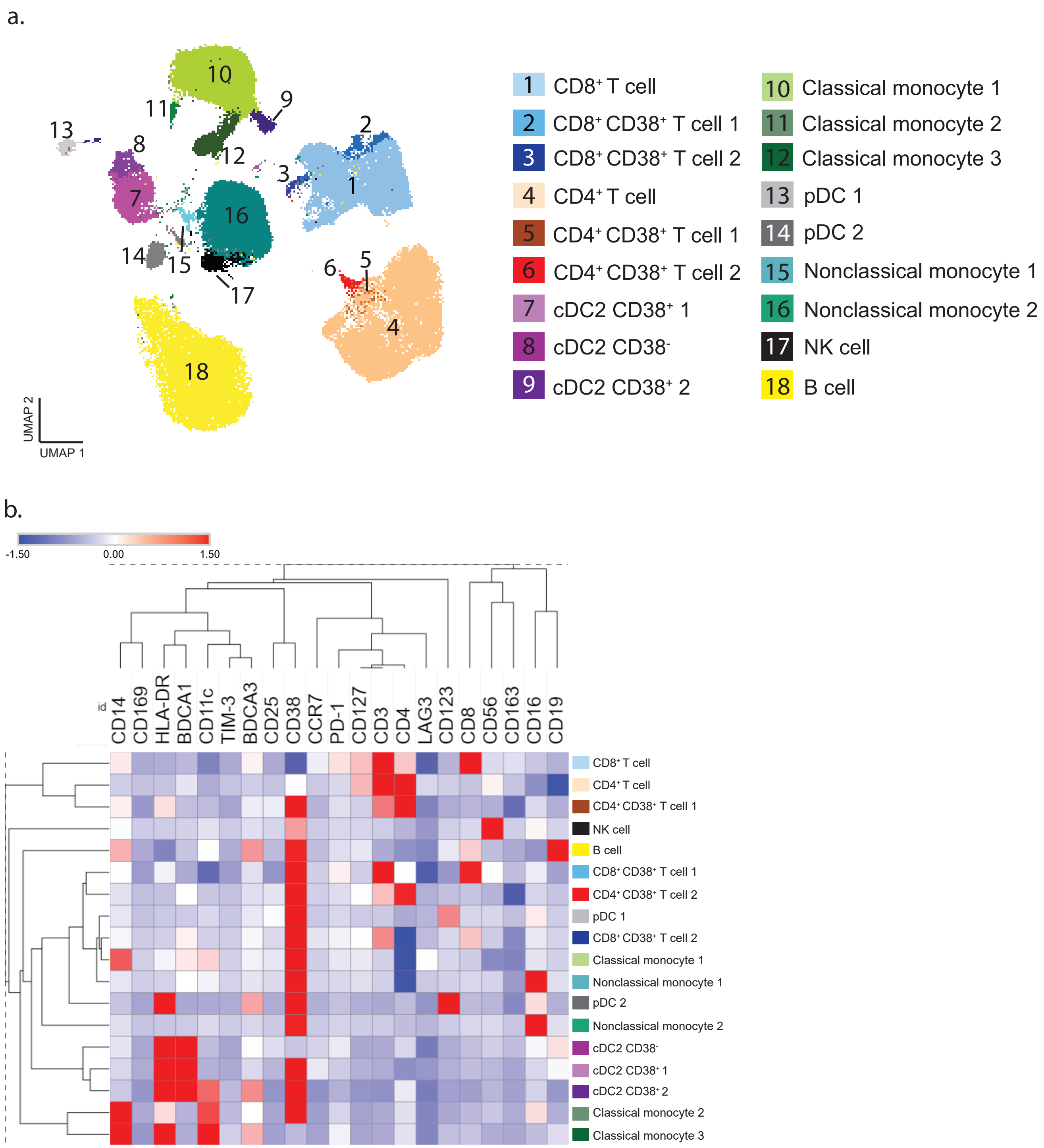

Supplementary Figure 5. *Unsupervised clustering of immune cells in healthy PBMCs*
a) UMAP projection of annotated clusters from healthy PBMCs. **b**) Generated heatmap of marker panel expression and hierarchical clustering for cluster annotation in PBMCs. Blue denotes lower expression while red denotes higher expression of each marker within a cluster (top scale bar, left to right). Hierarchical clustering was performed by one minus Pearson correlation.
